# Prevalence and molecular characterization of G6PD deficiency in two *Plasmodium vivax* endemic areas in Venezuela: predominance of the African A-^202A/376G^ variant

**DOI:** 10.1186/s12936-015-1069-5

**Published:** 2016-01-11

**Authors:** Esmeralda Vizzi, Gilberto Bastidas, Mariana Hidalgo, Laura Colman, Hilda A. Pérez

**Affiliations:** Laboratorio de Biología de Virus, Centro de Microbiología y Biología Celular, Instituto Venezolano de Investigaciones Científicas, Apdo 21827, Caracas, 1020-A Venezuela; Laboratorio de Inmunoparasitología, Centro de Microbiología y Biología Celular, Instituto Venezolano de Investigaciones Científicas, Apdo 21827, Caracas, 1020-A Venezuela; Departamento de Salud Pública, Facultad de Ciencias de la Salud, Universidad de Carabobo, Valencia, Edo. Carabobo Venezuela

**Keywords:** G6PD deficiency, Mutation analysis, Venezuelan population

## Abstract

**Background:**

Glucose-6-phosphate dehydrogenase (G6PD) deficiency causes acute haemolytic anaemia triggered by oxidative drugs such as primaquine (PQ), used for *Plasmodiumvivax* malaria radical cure. However, in many endemic areas of vivax malaria, patients are treated with PQ without any evaluation of their G6PD status.

**Methods:**

G6PD deficiency and its genetic heterogeneity were evaluated in northeastern and southeastern areas from Venezuela, Cajigal (Sucre state) and Sifontes (Bolívar state) municipalities, respectively. Blood samples from 664 randomly recruited unrelated individuals were screened for G6PD activity by a quantitative method. Mutation analysis for exons 4–8 of *G6PD* gen was performed on DNA isolated from G6PD-deficient (G6PDd) subjects through PCR–RFLP and direct DNA sequencing.

**Results:**

Quantitative biochemical characterization revealed that overall 24 (3.6 %) subjects were G6PDd (average G6PD enzyme activity 4.5 ± 1.2 U/g Hb, *moderately deficient, class III*), while DNA analysis showed one or two mutated alleles in 19 of them (79.2 %). The *G6PD* A-^202A/376G^ variant was the only detected in 17 (70.8 %) individuals, 13 of them hemizygous males and four heterozygous females. Two males carried only the 376A → G mutation. No other mutation was found in the analysed exons.

**Conclusions:**

The G6PDd prevalence was as low as that one shown by nearby countries. This study contributes to the knowledge of the genetic background of Venezuelan population, especially of those living in malaria-endemic areas. Despite the high degree of genetic mixing described for Venezuelan population, a net predominance of the mild African *G6PD* A-^202A/376G^ variant was observed among G6PDd subjects, suggesting a significant flow of *G6PD* genes from Africa to Americas, almost certainly introduced through African and/or Spanish immigrants during and after the colonization. The data suggest that 1:27 individuals of the studied population could be G6PDd and therefore at risk of haemolysis under precipitating factors. Information about PQ effect on G6PDd individuals carrying mild variant is limited, but since the regimen of 45 mg weekly dose for prevention of malaria relapse does not seem to be causing clinically significant haemolysis in people having the *G6PD* A-variant, a reasoned weighing of risk–benefit for its use in Venezuela should be done, when implementing public health strategies of control and elimination.

## Background

Glucose-6-phosphate dehydrogenase (G6PD) deficiency is an erythro-enzymopathy, recessive and one of the most common X-linked hereditary genetic defects, due to mutations in the *G6PD* gene, which cause functional variants with many biochemical and clinical phenotypes, affecting about 400 million people worldwide [1, 2]. The related clinical manifestations range from haemolytic anaemia to favism, neonatal jaundice or rarely congenital non-spherocytic haemolytic anaemia [3, 4]. In general, an acute haemolytic attack can occur after taking certain oxidative drugs, such as primaquine (PQ), used in both the radical cure of *Plasmodiumvivax* malaria and the presumptive anti-relapse therapy in people with extensive exposure to *P. vivax*.

Although globally the estimated number of malaria cases decreased from 227 million in 2000 to 198 million in 2013, it remains the most important infectious disease in tropical and sub-tropical areas of the world [5]. Implementation of effective control, prevention, diagnosis, and treatment practices reduced by 47 % the malaria mortality rates worldwide between 2000 and 2013 in all age groups, 54 % in the WHO African Region and 53 % in children under 5-years of age. However, this disease caused 584,000 deaths worldwide (range 367,000–755,000) during 2013 [5].

In the Americas, a substantial reduction (>75 %) in the incidence of microscopically confirmed malaria cases was reached in all endemic countries between 2000 and 2013, with the exception of Guyana and Venezuela, for which a significant increase in malaria morbidity was described during the same period [5]. In Venezuela, 371,473 malaria cases were recorded during the last decade, while vivax malaria accounted for 82 % of all cases, followed by *Plasmodium falciparum* (16 %), *Plasmodium malariae* (<1 %), and *Plasmodium vivax/falciparum* mixed (1.4 %) infections [6]. In this country the parasitic annual index amounted to 15.1/1000 inhabitants in 2014, showing an increase of 16 % in the last decade [7], Bolívar, Amazonas and Sucre states being among the Venezuelan regions with higher malaria incidence (63.3, 16.9 and 12.5 %, respectively) [6].

The elimination of reservoirs of infection and reduction of transmission rates are crucial tasks to the success of any malaria eradication programme [2, 8]. PQ treatment is currently the only licensed therapy active against the latent liver stages of *P. vivax* and the only drug with activity against the mature transmission stages of all *Plasmodium* species [8, 9]. However, the main safety concern for PQ administration is the risk of acute haemolytic anaemia (AHA) in G6PD-deficient (G6PDd) individuals, who are uniquely vulnerable to oxidative stresses, as their erythrocytes do not have alternative pathways for G6PD-dependent, reduced nicotinamide adenine dinucleotide phosphate (NADPH) production, essential to maintain their two main anti-oxidant defenses, reduced glutathione and catalase [10]. Tafenoquine, a most advanced product and putative successor of PQ that recently completed phase 2 clinical trials, has been considered to be used as a single-dose radical cure therapy against *P. vivax*, but the haemolytic risk with this long-acting 8-aminoquinoline remains in force [11]. Intravascular haemolysis caused by PQ can vary from mild to severe in G6PDd individuals, but the severity of this event seems to be related to PQ dosing and the variant of the G6PD enzyme [9, 11]. PQ should not be administered to patients with vivax malaria without previous evaluation of their G6PD status. Haemolytic events associated with PQ use have been described in G6PDd individuals in LA and the Caribbean [12]. Increased risk for malaria-related transfusions and death triggered by PQ-induced haemolysis were described in G6PDd subjects from Brazilian Amazon, a region accounting for 99.8 % of the registered cases of malaria in Brazil [11]. One pediatric case of haemolysis was described in a traveler infected with malaria and treated with PQ, returning from an endemic area in Venezuela [13].

G6PD deficiency is still considered the most common of all clinically significant enzyme defects in human biology [3]. G6PD deficiency prevalence depends on the region and ethnic group and it is highly prevalent in areas historically exposed to *Plasmodium* infections, in agreement of selection by malaria [14], although it is more common in African and Asian than in European and American people [2]. A geostatistical model-based map has predicted an overall allele frequency of 8 % for G6PD deficiency, widespread across malaria-endemic regions, with lowest frequencies in the Americas and highest in sub-Saharan tropical Africa and Arabian Peninsula [2].On the other hand, most of the countries of Latin America (LA) presenting cases of PQ-induced haemolysis show a higher prevalence of G6PDd when compared to other countries [12].

Over 400 different biochemical variants of G6PD have been described globally [10, 15, 16]. The *G6PD* locus shows a considerable degree of genetic heterogeneity and at least 186 distinct alleles involving mutations leading to single amino acid substitutions or deletions, scattered throughout the entire coding and non-coding region, have been identified [3, 15, 17]. Most of the variants occur sporadically, although some, such as the *G6PD* Mediterranean and the *G6PD* A-^202A/376G^ variants, exist with an increased frequency in certain populations [18, 19]. For example, *G6PD* A-^202A/376G^ is distributed in relatively homogeneous manner in Africa and the Americas. Although some studies carried out in regions of South and Central America have previously shown heterogeneity of the G6PD variants [20–22], the *G6PD* A-^202A/376G^ has been described as the genetic variant most broadly distributed across LA, present in 81.1 % of the deficient individuals surveyed [12]. This variant is due to a G → A transition at nucleotide position 202, which causes a Val → Met replacement at amino acid position 68 of the protein, that is carried in combination with a A → G substitution at nucleotide 376, corresponding to a change Asn → Asp at amino acid position 126. It determines a mild form of G6PD deficiency. Instead, the *G6PD* Mediterranean^563T^, a C → T substitution leading to an amino acid change Ser → Phe at position 188, responsible for a severe form of G6PD deficiency, is widely distributed across southern Europe, the Middle East, through Iran, Afghanistan, Pakistan, and much of western India [2, 23]. However, more information is needed on the distribution of G6PD-deficiency variants in the world, as well as more affordable tests to identify at-risk individuals, particularly in malaria-endemic countries.

To date, limited studies have been undertaken to ascertain the prevalence of G6PD deficiency in Venezuelan people [24–27]. One of them reported deficiency of 2 % in subject blood donors living in Caracas and 11.5–13.3 % in Afro-descendant people of Tapipa (Miranda state) [24]. Another study conducted in Bolívar state found 5.3 % of deficiency in individuals with suspected malaria [25]. But information about the molecular heterogeneity of the G6PD deficiency in Venezuela is not currently available.

In this study the prevalence rate of G6PD enzymatic activity was evaluated and the *G6PD* genetic variant identified in apparently healthy individuals from northeastern and southeastern regions of Venezuela to assess the rate and genetic basis of G6PD deficiency in endemic areas of vivax malaria.

## Methods

### Subjects and samples

**Fig. 1:**
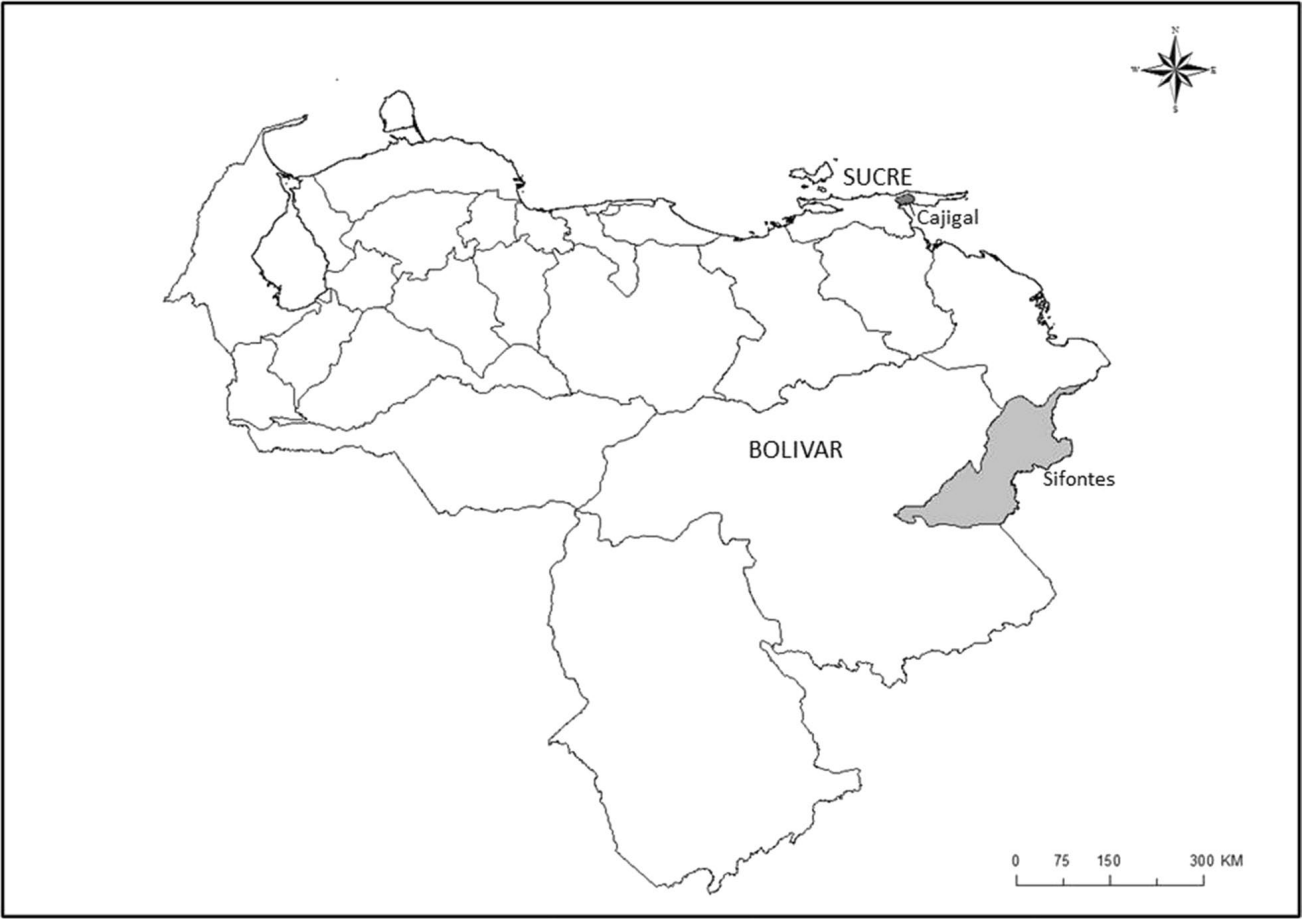
Map of Venezuela showing both municipalities included in the study of prevalence of G6PD deficiency. People studied were living in two malaria endemic regions of Venezuela, Sifontes municipality, in the southeast of Bolívar state, and Cajigal municipality, in the northeastern coastal plains of Sucre state, along the Caribbean

Blood samples from 664 randomly recruited unrelated individuals were collected during the years 2005 and 2006, of which 316 were females and 348 males. The sample represented all of those willing to do the screening and there was thus no bias in terms of gender (being females and males equally represented), age and ethnic origin. All of them were born in Venezuela, had a median age of 24 years (range 1–109 years), represented 1–2 % of the total population in the areas assessed and were living in two of the regions of Venezuela with high malaria incidence: Sifontes municipality (332 individuals), located in the southeast of Bolívar state, near the lowland rain forest and savannas of Guayana, where the most of the population (32.621 inhabitants) is exposed at high risk to malaria due to economic activities, such as agriculture, mining and forest exploitation, and Cajigal municipality, Sucre state (332 subjects), in the northeastern coastal plains, along the Caribbean Sea, largely composed of mangroves, herbaceous and woody swamps, where the population (18.942 inhabitants) lives mainly of fishing, subsistence agriculture, and tourism (Fig. 1). Both areas show annual mean temperature around 24–28 °C and rainfalls that are more directly influenced by the Atlantic inter-tropical convergence zone. A written informed consent was obtained from each participant authorizing the collection of a 4-ml whole blood sample in ethylenediaminetetraacetic acid (EDTA) as anticoagulant, which was stored at 4 °C during the fieldwork, placed in liquid nitrogen within 24 h of collection and kept frozen until analysis.

### Biochemical analysis of G6PD deficiency

The blood samples were screened for G6PD enzymatic activity using a quantitative method (NeoLISA G6PD assay, Interscientific^®^ Corp, FL, USA) through the haemoglobin normalization procedure, following manufacturer’s instructions, and the values used to determine G6PD activity were expressed in U/g Hb (units per gram haemoglobin). The controls used were supplied by the manufacturer in three levels of G6PD activity (normal = 14.3 U/g Hb, intermediate = 4.7 U/g Hb and deficient = 1.3 U/g Hb) at 37 °C, and the cut-off point was 7.14 U/g Hb to define a subject as G6PDd on the basis of adjusted male median G6PD activity, as recommended by Domingo et al. [28]. The rate of NADPH generation was spectrophotometrically measured at 570 nm (Titertek Multiskan^®^, Phoenix Equipment Inc, USA). Samples of subjects with reduced activity were tested for *G6PD* gene mutations.

### Molecular analysis of *G6PD* variants

Genomic DNA was extracted from G6PDd whole blood samples by using QIAamp DNA Blood Mini kit (Qiagen^®^, GmbH, Germany), according to manufacturer’s recommendations, and used as templates to search for the more common *G6PD* genetic variants whose mutations are located within the exons 4–8 of *G6PD* gen. A polymerase chain reaction (PCR) using four pairs of previously described oligonucleotide primers [29, 30] and cycling conditions showed in Table 1, were applied to amplify the corresponding regions of exons 4–8 in a thermal cycler Eppendorf MasterCycler Personal (Eppendorf, Hamburg, Germany). PCR products were analysed by agarose gel electrophoresis and ethidium bromide staining, and cleaved to perform the restriction fragment length polymorphism (RFLP) analysis with 5 U of *Nla*III, *Fok*I and *Mbo*II endonucleases, in accordance with procedures previously described [31, 32], and under conditions recommended by the manufacturer (New England BioLab Inc, Beverly, MA, USA) to identify the 202G → A, 376A → G, 563C → T, and 844G → C substitutions (Table 1). Control DNA samples, carrying the appropriate restriction site, were run along in each digestion set. Digested products were separated on 10 % polyacrylamide gel electrophoresis for 1 h at 100 V and visualized by ethidium bromide staining. In addition, in order to search other nucleotide substitutions located into the amplified exons (185C → A from exon 4, 542A → T, 592C → T, 593G → C, 634A → G, 637G → T and 680G → A from exon 6/7, 820G → A, 835A → T, 854G → A and 871G → A from exon 8) and to confirm RFLP analysis results, all PCR products were further analysed by automated direct DNA sequencing in both directions using BigDye Terminator Cycle Chemistry (Macrogen^*®*^ Inc, Korea).

**Table 1:** PCR/RFLP conditions used to identify the *G6PD* genetic variants of G6PDd unrelated individuals in the present study

Mutations identified by PCR/RFLP^a^	Exon	Primer oligonucleotide sequence (5′–3′)	Amplicon size^b^ (bp)	References	RFLP pattern (fragment size in bp)		
					RE^c^	Wild-type	Mutant^d^
202G → A	4	GTGGCTGTTCCGGGATGGCCTTCTG CTTGAAGAAGGGCTCACTCTGTTTTG	109	29	*Nla*III	109	63, 46
376A → G	5	CAGTACGATGATGCAGC CAGGTAGAAGAGGCGGT	90		*Fok*I	90	58, 32
563C → T	6/7	ACTCCCCGAAGAGGGGT CCAGCCTCCCAGGAGAGA	542	30	*Mbo*II	25, 26, 119,377	25, 26, 100, 119, 277
844G → C	8	GGAGCTAAGGCGAGCTC CATGCTCTTGGGGACTG	230		*Nla*III	11, 34, 75,110	11, 28, 47, 34,110

*RFLP* restriction fragment length polymorphism analysis, *bp* base pairs, *RE* restriction endonuclease used for RFLP

^a^The remaining mutations located in the exons 4–8, specifically the 185C → A, 542A → T, 592C → T, 593G → C, 634A → G, 637G → T, 680G → A, 820G → A, 835A → T, 854G → A and 871G → A, were studied by DNA sequencing

^b^Cycling conditions used in the PCR were as follows: 40 cycles at 94 °C for 1 min, 60 °C (for exon 4 and 8) or 56 °C (for exon 5) or 58 °C (for exon 6/7) for 30 s, and 72 °C for 40 s. A final elongation at 72 °C for 7 min was added

^c^Restriction endonuclease digestion carried out with 5 U of enzyme at 37 °C for 3 h

^d^ *Nla*III*, Fok*I or *Mbo*II recognition site is created by the mutation

### Statistical analysis

Data were analysed for the comparisons of variables using 2 × 2 tables with χ^2^ test, or Fisher’s exact test (two-tailed, 95 % confidence intervals) when the size sample was less than 5 (Epi Info™ 7.1.4.0, CDC Atlanta, GA, USA). Mean, median (both stratified by gender), standard deviation and range of G6PD enzymatic activities were calculated to determine reference values in normal and deficient subjects. Student’s test was applied for comparisons of variable values. Tests were considered significant when p < 0.05.

## Results

### Prevalence of G6PD deficiency

The biochemical characterization revealed that 24/664 (3.6 %) subjects were G6PD-deficient as their G6PD activity level was less than 60 % of the adjusted male median value (Table 2). The overall prevalence of G6PD deficiency was higher in subjects from Cajigal (Sucre state) than from Sifontes municipality (Bolívar state) (6 vs 1.2 %, p = 0.001), and the median age of the G6PDd subjects was 23 (± 21.5) years. Male to female ratio was approximately 2:1, but no statistically significant difference was observed (p = 0.11), being the G6PD deficiency rate among males 4.7 % (*n* = 16/337) and among females 2.4 % (*n* = 8/327), considering 5 % standard error and 95 % confidential intervals (Table 2).

**Table 2:** Prevalence of G6PD deficiency in Venezuelan individuals from Sifontes and Cajigal municipalities and *G6PD* gene alleles from subjects with biochemical deficiency

Geographical setting	Gender	TOTAL	G6PD determination results				
			Biochemical test*		DNA analysis^(^**^)^		
			Normal^(a)^	G6PDd^(#, b)^	Wild-type allele	202G → A allele	376A → G allele
Cajigal municipality (Sucre state) *n* = 332	F	163	156 (95.7)	7 (4.3)	3	4	4
	M	169	156 (92.3)	13 (7.7)	1	11	12
Sifontes municipality (Bolívar state) *n* = 332	F	164	163 (99.4)	1 (0.6)	1	0	0
	M	168	165 (98.2)	3 (1.8)	0	2	3
		664	640 (96.4)	24 (3.6)	5 (20.8)	17 (70.8)	19 (79.2)

Data are in number (percentage) of cases for each condition

*F* females, *M* males, *G6PDd* G6PD-deficient subjects

^***#***^ *p* = 0.001 by Fisher’s exact test for the comparison of G6PD deficiency prevalence among subjects of Cajigal vs. Sifontes municipality

* The biochemical test used was a NeoLiSA G6PD assay (Interscientific^®^, Hollywood, USA). *p* < 0.0001 by Student’s test for comparison of mean G6PD enzyme activity among normal and G6PDd subjects

** DNA analysis by PCR/RFLP and sequencing of the exons 4–8 of *G6PD* gene from subjects with biochemical deficiency. The other alleles, which were studied by sequencing (185C → A, 542A → T, 592C → T, 593G → C, 634A → G, 637G → T, 680G → A, 820G → A, 835A → T, 854G → A and 871G → A) resulted wild-type

^a^Mean G6PD enzyme activity of 12.9 ± 3.8 U/g Hb [defined as *Class IV *(in 84 %) and *V* (in 2 %) (data not shown)], according to WHO criteria (33, 34)

^b^Mean G6PD enzyme activity of 4.5 ± 1.2 U/g Hb (defined as *moderately deficient enzyme, Class III*, according to WHO criteria [33, 34]. *p* = 0.007 among G6PDd males and females compared by Student’s test

All G6PDd subjects detected were asymptomatic. The mean G6PD enzyme activity was of 4.5 ± 1.2 U/g Hb (ranging from 2.6 to 6.2 U/g Hb), significantly lower than that observed in normal subjects (12.9 ± 3.8 U/g Hb, *p* < 0.0001). This G6PD deficiency could be classified as moderately deficient enzyme, class *III,* according to the World Health Organization (WHO) criteria [31, 32]. A mean value of G6PD enzyme activity significantly higher was observed among G6PDd females than in males (5.5 vs 4.1 %, *p* = 0.007).

### Identification of *G6PD* gene variants in the deficient subjects identified

The PCR–RFLP analysis showed that 17 (70.8 %) out of 24 G6PDd subjects with deficiency detected biochemically carried the mutated allele 202G → A and 19 (79.2 %) the substitution 376A → G. These results were all confirmed by DNA sequencing. No mutation was detected in five (20.8 %) out of 24 G6PDd subjects, as well as none of the additional mutations studied by DNA sequencing mapping into the exons 4–8. The information is summarized in Table 2.

The analysis showed that 17 (70.8 %) out of 24 subjects were carriers of both 202G → A and 376A → G mutated alleles, that define the *G6PD* A-variant of African origin. Thirteen (76.5 %) of them were hemizygous (males) and four (23.5 %) heterozygous (females) for each mutation. In addition, the 376A → G allele was detected in hemizygosis in two males (Table 2).

## Discussion

Malaria remains a global health problem. In Venezuela malaria is associated with socio-economic problems and failures in preventive measures and social protection actions. The wide distribution in the world of the G6PD deficiency further hampers malaria control efforts. Epidemiological studies to evaluate the distribution of this enzyme defect, particularly in regions with the highest risk of transmission of *P.vivax,* allowing implementation of PQ-based malaria radical cure programmes, as well as for the evaluation of new, more effective anti-parasite drugs, potentially able to cause haemolysis, and of appropriate measures of tolerability and risk of severe adverse events, are needed. Moreover, the application of G6PD deficiency tests may dramatically increase the benefits of PQ therapy by permitting the application of relatively higher dose than the current standard regimen, and shortening treatment length.

There is currently no practical point-of-care field test for the detection of G6PDd subjects, so from a public health perspective uncertainty remains about the best procedures to follow. Studies of cost-effectiveness and budget impact for health services are recommended to support the incorporation of these tests into control programmes aimed at malaria elimination in endemic countries.

Because the haemolytic risk of PQ-based regimens in malaria-endemic regions depends also on the severity of *G6PD* mutations involved in the deficiency, *G6PD* genetic testing could be useful when the benefits of PQ treatment outweigh the risk in patients living in these areas. Moreover, G6PD biochemical tests could fail to detect a fraction of heterozygous subjects, although these have a bigger population of G6PD-normal erythrocytes, and therefore are unlikely to develop a serious haemolytic attack. The determination of the *G6PD *genetic variant allows more accurate prediction of the risk of haemolysis.

In this work the prevalence of G6PD deficiency was evaluated in subjects living in two of the most prevalent malaria regions of Venezuela, and for the first time the African *G6PD* A-^202A/376G^ genetic variant involved in such enzyme deficiency was identified and confirmed at the molecular level as predominant.

The overall prevalence of G6PD deficiency (3.6 %) observed in the analysed Venezuelan population was low, although it was significantly higher in the sample from Sucre state than that from Bolívar state, which is probably due to higher prevalence of African heredity in the north coastal area of Venezuela, as a result of slave immigration during colonial times [35]. This rate of G6PD deficiency is similar to that shown on a map modelling prevalence (>5 %) in Venezuela proposed by other authors [2]. The higher frequency of biochemical deficiency of G6PD observed in males than in females could be due to a skewed X-chromosome inactivation (or lyonization) in females, which determines the survival and growth of G6PD normal cells in G6PD heterozygous females, which could have been missed by the biochemical procedure but not by genetic testing.

The results obtained here suggest that 1:27 individuals of the studied population could be G6PDd and therefore at risk of haemolysis in the presence of precipitating factors. Low G6PDd subjects frequencies (<10 %) have been described in other LA countries, such as Argentina, Bolivia, Mexico, Perú, and Uruguay, but higher in the Caribbean islands, Guianas, Pacific coastal regions of Colombia and Ecuador, and part of the Brazilian Atlantic coast, which are areas that received the greatest contribution of Africans during the slave trade [12, 23].

Two mutations were detected in most (71 %) of subjects with biochemically determined deficiency, particularly the 202G → A and the 376A → G, defining together the variant *G6PD* A-^202A/376G^, widely distributed in Africa where it seems to confer resistance to falciparum malaria [19, 36], and identified under other names in Spain, Mexico, Italy, and many other parts of the world [12, 37]. High prevalence of this *G6PD* variant has been reported in Brazil, Mexico, Cuba and Honduras [20–22, 38], but a great diversity of variants has been described and spread across American, European and Asian countries [12, 19, 23]. Although in this study four regions were studied spanning five exons of the *G6PD* gene, which include the most common mutations described in G6PDd subjects of LA countries, it is not possible to exclude those mutations located in other gene regions which can be involved in the deficiency. Even so, it is noteworthy the relatively homogeneous molecular base of the G6PD deficiency found in the analysed sample of Venezuelan population, contrasting with the high heterogeneity reported in several studies that revealed a substantial number of *G6PD* variants in LA populations, such as the *G6PD* A-^376G/968C^, *G6PD* Santamaría^376G/542T^, *G6PD* Seattle^844C^, *G6PD* Mediterranean^563T^, and *G6PD* Union^1360T^ described in Mexico, Brazil and Cuba [20–22].

Although a systematic study from individuals living in other Venezuelan regions would be desirable to better understand the genetic background in the country, the presence of only two G6PD alleles, 202G → A and the 376A → G, in the people studied can be justified. The current Venezuelan population is the result of an intense racial admixture including diverse components, such as Amerindians, Europeans and Africans [35]. However, the mutated alleles 202G → A and the 376A → G may have been introduced in the country during the admixture process predominantly by the African contribution, mainly from the sub-Saharan region, which occurred in the 16th, 17th and 18th centuries, the period when the slave trade was most active, but also later from the Caribbean islands [35, 39, 40]. As described by some authors, genetic heredity of African populations in Venezuela is basically concentrated in the African-derived towns, the entire coastal area of the country. Their geographic distribution was directly linked to the location of the agricultural plantations during the slave trade, and the distribution pattern of genes of the Colonial era reflect a very limited gene flow due to the isolation and endogamy typical of this population, with low or no mixture with Europeans, while the Amerindian lineages are almost non-existent [35]. Previous studies from different areas of this continent confirmed the virtual absence or a very low frequency of G6PDd subjects in Amerindian populations, including those of Amazonas state in Venezuela [12, 41, HA Pérez, pers comm]. Interestingly, *G6PD* A-^202A/376G^ is the most common variant described among the Spanish population [37], and considering the important contribution that Spanish immigration had on the admixture process in Venezuela, a significant gene flow from Africa to Europe and America through Spain cannot be excluded.

There has been considerable confusion about the drugs that are capable of producing haemolytic anaemia in patients with G6PD deficiency. The risk of serious harm in any given population exposed to PQ therapy remains a concern. Although a mild methaemoglobinaemia typically occurs with normal PQ dosing, it is probable that other precipitating factors, such as infections or treatment with other drugs than PQ, can cause a slight shortening of the red cell lifespan in G6PDd patients and elicit a haemolytic episode. African A- has frequently been considered a variant associated with mild disease. In subjects carrying a G6PD A- variant, known to be a moderately deficient enzyme, the red cells retain more than 10 % of residual G6PD activity, mostly present in reticulocytes and younger erythrocytes [42]. As a consequence of this residual activity, an acquired tolerance to daily PQ dosing can be developed in these patients, who are relatively resistant to PQ-induced haemolysis [43]. But contrary to current perception, the G6PD A- variant cannot be considered ‘mild’, because a significant haemoglobin drop has been observed in studies with African patients using anti-malarial preparations containing dapsone [44] or after administration of a gametocytocidal drug combination containing PQ and an artemisinin, in 40 % of children carrying the G6PD A- variant in Tanzania [45]. It cannot exclude that the A- variant may be involved in severe haemolytic crisis.

On the other hand, the current Food and Drug Administration (FDA)-approved PQ dose, to prevent relapse by *P.vivax* and *P.ovale* by killing liver stage hypnozoites, is 15 mg daily for 14 days (adult dose). This dose, used in combination with 25 mg of chloroquine, remains the recommended treatment for radical cure of adults with vivax malaria, and is implemented in Venezuela at present. However, *P.vivax* strains acquired in some parts of the world require a higher dose of PQ to prevent relapse. To achieve reliable eradication of parasites, the CDC has recommended an increase in dose from 15 to 30 mg daily for 14 days for adults [46]; although expert opinion and clinical trial data support this recommendation, the 30-mg daily dose is not FDA approved. More recently, a regimen of a 45-mg weekly dosing of PQ (adult dose) for eight weeks, which seems to be effective treatment against strains of *P.vivax* not killed by standard therapeutic regimens, has been recommended to treat individuals with a partial G6PD deficiency by a WHO expert committee [9, 19, 47, 48]. Although the data to support efficacy against malaria are limited, this dose has been associated with a limited and clinically apparent haemolytic anaemia in moderately G6PD-deficient subjects [48], but these findings do not conclusively indicate that non-specific administration of PQ is safe. Currently there is still no international agreement on the frequency and type of G6PD deficiency, justifying the suspension of the use of PQ in the treatment of *vivax* malaria, but the implementation of drugs into malaria eradication programmes should go along with a more thorough assessment of the clinical burden of G6PD condition in each region. Because Venezuela does not carry out the test for G6PDd before starting treatment with PQ, its use at higher doses should be applied only after a careful risk/benefit assessment in a known G6PDd individual and under strict medical supervision.

## Conclusion

The prevalence rate of G6PD deficiency was as low as that one shown by nearby countries, and the molecular base was relatively homogeneous. Understanding the G6PD deficiency and its diversity are key issues to analysing evidence of PQ safety in malaria-endemic areas. This is the first survey conducted to identify the genetic variant involved in G6PD deficiency in Venezuelan subjects, and it represents a contribution to the knowledge of the genetic structure of this population.

## Abbreviations

G6PD: Glucose-6-phosphate dehydrogenase
PQ: Primaquine
G6PDd: G6PD-deficient
PCR: Polymerase chain reaction
RFLP: Restriction fragment length polymorphism
AHA: Acute haemolytic anaemia
NADPH: Nicotinamide adenine dinucleotide phosphate
LA: Latin America
EDTA: Ethylenediaminetetraacetic acid
U/g Hb: Units per gram haemoglobin
CDC: Center for disease control and prevention
WHO: World Health Organization
FDA: Food and Drug Administration
